# Exosome therapy for knee osteoarthritis: a network meta-analysis based on rat models

**DOI:** 10.1093/stcltm/szag069

**Published:** 2026-08-18

**Authors:** Yixiang Zhao, Haochuan You, Xiuyuan Wang, Xiping Dou, Jiangying Zhang, Yuzhe He, Lei Chen, Fei Huang, Yayi Xia

**Affiliations:** Department of Orthopedics, Lanzhou University Second Hospital, Lanzhou, 730030, China; Department of Orthopedics, Lanzhou University Second Hospital, Lanzhou, 730030, China; Department of Orthopedics, Lanzhou University Second Hospital, Lanzhou, 730030, China; Department of Orthopedics, Lanzhou University Second Hospital, Lanzhou, 730030, China; Department of Orthopedics, Lanzhou University Second Hospital, Lanzhou, 730030, China; Department of Orthopedics, Lanzhou University Second Hospital, Lanzhou, 730030, China; Department of Orthopedics, Lanzhou University Second Hospital, Lanzhou, 730030, China; Department of Orthopedics, Lanzhou University Second Hospital, Lanzhou, 730030, China; Department of Orthopedics, Lanzhou University Second Hospital, Lanzhou, 730030, China

**Keywords:** knee osteoarthritis, exosomes, network meta-analysis, rat model, cartilage repair

## Abstract

**Objective:**

To compare the therapeutic effects of exosomes from different cell sources and doses in rat knee osteoarthritis models using network meta-analysis, and to identify potential optimal strategies for preclinical optimization and clinical hypothesis generation.

**Methods:**

A systematic search of PubMed, Web of Science, Embase, and Scopus identified 27 eligible randomized controlled trials (456 rats). Traditional random-effects meta-analysis and frequentist network meta-analysis were conducted. Treatments were ranked using SUCRA values.

**Results:**

A total of 27 studies were included, comprising 456 rats. Traditional meta-analysis revealed that exosome therapy significantly reduced joint cartilage histopathological damage (reduced OARSI score: SMD = −3.78, 95% CI: −4.76, −2.80), promoted type II collagen synthesis (SMD = 3.41, 95% CI: 1.87, 4.95), and suppressed the expression of the inflammatory cytokine IL-1β (SMD = −3.26, 95% CI: −4.08, −2.44). Network meta-analysis further indicated that under high-dose conditions (≥100 μg), exosomes derived from human amniotic fluid stem cells (hAFSCs-Exo) and human umbilical cord mesenchymal stem cells (hUCMSCs-Exo) ranked highest in relative efficacy for improving the OARSI score. hUCMSCs-Exo also demonstrated the most potent anti-inflammatory effect by reducing IL-1β. Overall, exosome therapy exhibited a dose-related trend in efficacy, with high-dose transplantation showing higher probabilistic SUCRA rankings across most outcome measures. However, assessment of publication bias revealed marked asymmetry; after trim-and-fill correction, the effect size for type II collagen expression decreased from SMD = 3.41 to 1.26, suggesting that the magnitude of the treatment effect may have been overestimated. This overestimation reduces the certainty of the observed treatment effects. Despite the presence of publication bias and methodological limitations, the main conclusions remained statistically robust after trim-and-fill correction.

**Conclusion:**

Current evidence from rat models suggests that exosome therapy may substantially ameliorate the pathological progression of KOA, with exosomes derived from early developmental tissues, particularly hAFSCs-Exo and hUCMSCs-Exo, showing favorable chondroprotective and anti-inflammatory effects in the probabilistic ranking when administered at high doses. Nevertheless, the findings from publication bias and trim-and-fill analyses indicate that the existing effect sizes are likely overestimated. These results require confirmation in larger-scale, methodologically more rigorous preclinical studies.

## Graphical Abstract

**Graphical Abstract:**
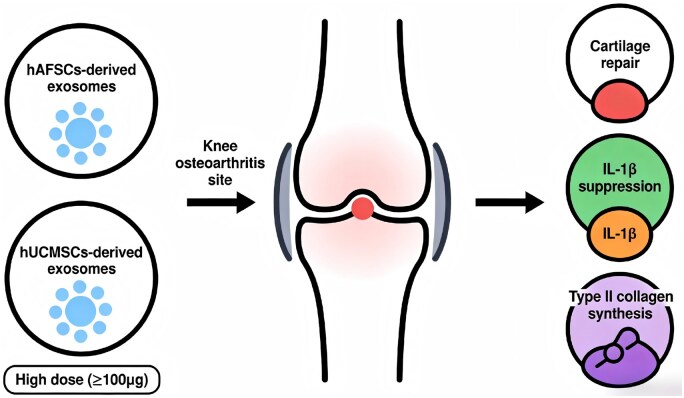

Significance statement This study addresses the challenge of identifying the most effective exosome-based therapy for knee osteoarthritis, a debilitating joint disease with limited regenerative treatment options. For the first time, a network meta-analysis was used to comprehensively compare exosomes derived from different cellular sources and administered at varying doses. The findings reveal that exosomes originating from early developmental tissues showed a relatively higher probability of cartilage repair and anti-inflammatory effects in the SUCRA rankings, and higher doses were associated with better outcomes in the probabilistic ordering. Publication bias analyses (including funnel plot asymmetry and trim-and-fill correction) indicate that the observed effect sizes are likely overestimated, which reduces the certainty of these findings. This work provides comparative preclinical evidence on exosome sources and doses in rat models of knee osteoarthritis.

## Introduction

Knee osteoarthritis (KOA) is a chronic, disabling disorder driven primarily by progressive degeneration of articular cartilage. The number of affected individuals worldwide has reached 375 million and continues to increase.^1–4^ Current clinical treatments, including pharmacotherapy and surgery, can only relieve symptoms and cannot achieve repair of the damaged cartilage or reverse disease progression. Therefore, there is an urgent need for regenerative medicine strategies that promote tissue repair.

Exosomes derived from mesenchymal stem cells (MSCs), key mediators of paracrine signaling, have become a major focus because they can regulate chondrocyte metabolism, suppress inflammation, and facilitate repair. By avoiding potential risks associated with cell transplantation, they offer a new therapeutic avenue for KOA.^5–8^ Preclinical studies have demonstrated that exosomes can effectively slow osteoarthritis progression.^9^ However, existing studies are limited to direct comparisons of single strategies, with insufficient integrated evaluation of different exosome sources, doses, and delivery routes. Moreover, these studies often pool results across species, which reduces the reliability of the conclusions.^10–13^ Network meta-analysis can combine both direct and indirect evidence to compare and rank different interventions. Therefore, in this study, we combined conventional and network meta-analytic approaches based on rat KOA models to systematically evaluate the relative efficacy of different exosome-based treatment strategies, with the aim of preliminarily identifying potential directions for optimizing exosome clinical translation and providing a hypothesis-generating framework for future research.

## Methods

### Inclusion and exclusion criteria

The design, conduct, and reporting of this study strictly followed the Preferred Reporting Items for Systematic Reviews and Meta-Analyses (PRISMA) guidelines. Eligible studies were randomized controlled trials (RCTs). The population was limited to experimental rat KOA models. Interventions consisted of treatment with natural, unmodified exosomes, and studies were required to clearly report the exosome source, single or total transplantation dose (predefined as low: <40 μg; medium: 40-100 μg; high: ≥100 μg), and delivery route. Control groups included blank controls, vehicle controls, or other exosome intervention controls. The outcomes analyzed in this study were: (1) OARSI histological score, reflecting the structural integrity and degree of degeneration of articular cartilage; (2) type II collagen expression, reflecting the anabolic status of the cartilage extracellular matrix; and (3) levels of the inflammatory cytokine interleukin-1 beta (IL-1β), reflecting the intensity of the inflammatory response. These 3 endpoints were selected not only for their biological relevance but also because they were the most consistently reported in the included studies; other outcomes were not pooled quantitatively due to insufficient reporting. Studies were excluded if they were not RCTs, did not use rat models, employed engineered exosomes or exosomes combined with scaffolds, or failed to report key parameters adequately.

### Literature search and screening

We systematically searched PubMed, Web of Science, Embase, and Scopus up to October 1, 2025. The search strategy combined subject headings and free-text terms (eg, “exosome,” “knee osteoarthritis”), and reference lists were manually screened. Two investigators independently performed study screening and data extraction, with disagreements resolved by discussion or third-party arbitration. The detailed search strategy is provided in Table S1. Extracted data included basic study information, model details, intervention parameters, outcome data, and methodological quality.

### Risk of bias and statistical analysis

Risk of bias was assessed using the SYRCLE tool. For conventional meta-analysis, continuous outcomes were pooled using standardized mean differences (SMDs). Fixed- or random-effects models were selected according to heterogeneity (*I*^2^ statistic), and subgroup analyses were conducted based on exosome source, dose, and delivery route. Publication bias was evaluated using funnel plots and Egger’s/Begg’s tests. For network meta-analysis, an evidence network was constructed, and a consistency model was used to estimate relative treatment effects; inconsistency was examined using the node-splitting approach. Treatments were ranked using the surface under the cumulative ranking curve (SUCRA). All analyses were performed in R and STATA, and *P* < .05 was considered statistically significant. Detailed methods for risk-of-bias assessment and statistical analysis are provided in the Supplementary Material.

## Results

### Study selection and characteristics of included studies

After systematic searching and screening, 27 studies were included (Figure S1, see online supplementary material for a color version of this figure). A total of 456 rats were enrolled (mainly Sprague–Dawley and Wistar strains), and KOA was predominantly induced surgically (eg, ACLT). All interventions involved intra-articular injection of unmodified natural exosomes. The main sources were bone marrow MSC-derived exosomes (BMSCs-Exo, 12 studies), adipose MSC-derived exosomes (ADMSCs-Exo, 6 studies), human umbilical cord MSC-derived exosomes (hUCMSCs-Exo, 4 studies), and other sources (5 studies), including synovial fibroblast-derived exosomes (SFB-Exo), M2 macrophage-derived exosomes (M2D-Exo), human amniotic fluid stem cell-derived exosomes (hAFSCs-Exo), dendritic cell-derived exosomes (DC-Exo), and human urine-derived stem cell exosomes (hUSCs-Exo). Doses were categorized as low (<40 μg, 8 studies), medium (40-100 μg, 10 studies), and high (≥100 μg, 9 studies). Basic characteristics are shown in Table S2. Risk-of-bias assessment indicated that most studies provided insufficient reporting for key methodological domains such as random sequence generation and allocation concealment, resulting in an “unclear risk” rating (Figure S2, see online supplementary material for a color version of this figure).

### Meta-analysis results

#### OARSI score

**Figure 1.**
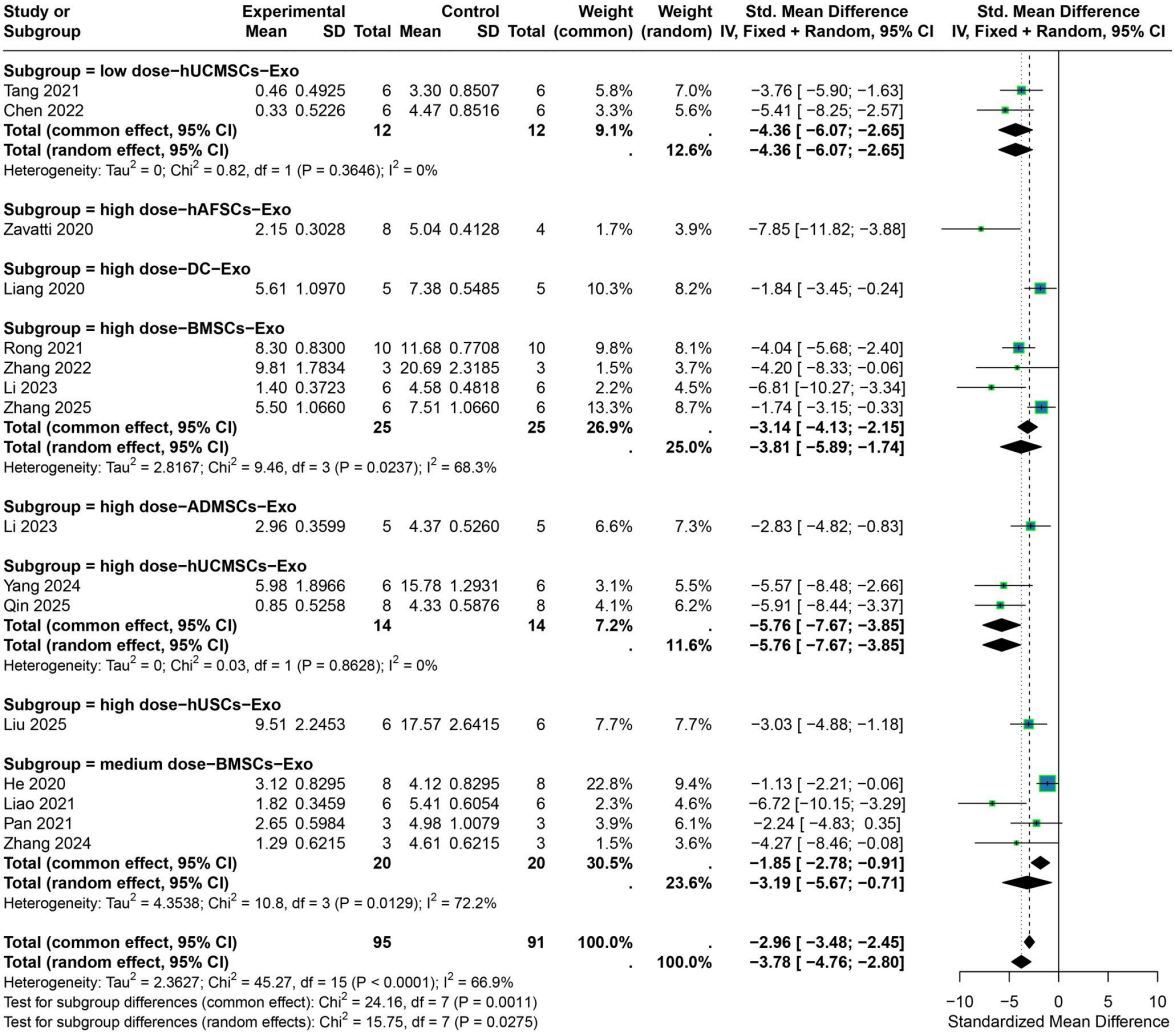
Conventional meta-analysis results for the OARSI score.

Sixteen studies reported OARSI scores. Conventional meta-analysis using a random-effects model (*I*^2^ = 66.9%, *P* < .0001) showed that exosome treatment significantly reduced the OARSI score of articular cartilage (SMD = −3.78, 95% CI: −4.76 to −2.80). Subgroup analyses revealed that, for exosomes from the same source, BMSCs-Exo significantly improved OARSI scores in both the high-dose group (SMD = −3.81) and the medium-dose group (SMD = −3.19), with a stronger effect at high dose; hUCMSCs-Exo showed a similar pattern, with a larger effect size at high dose (SMD = −5.76) than at low dose (SMD = −4.36). Under the same high-dose condition, efficacy differed across sources, with effect sizes ranked from highest to lowest as follows: hAFSCs-Exo (SMD = −7.85), hUCMSCs-Exo (SMD = −5.76), BMSCs-Exo (SMD = −3.81), hUSCs-Exo (SMD = −3.03), ADMSCs-Exo (SMD = −2.83), and DC-Exo (SMD = −1.84; Figure 1).

**Figure 2.**
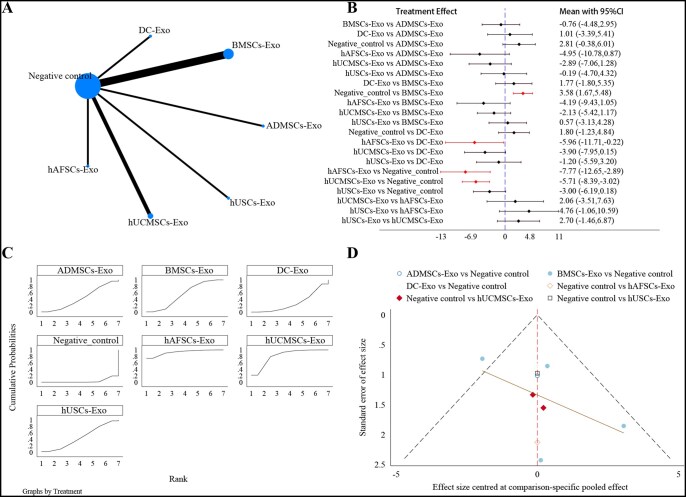
Effects of different exosome (Exo) types on improving the OARSI score at high dose. (A) Evidence network; (B) forest plot; (C) SUCRA ranking; (D) comparison-adjusted funnel plot.

To compare the effects of different exosome types on OARSI score improvement in rats with KOA, we conducted a network meta-analysis restricted to the high-dose condition (≥100 μg). The evidence network showed direct comparisons between exosome interventions and the negative control (Figure 2A). Inconsistency testing indicated no statistically significant local or global inconsistency (all *P* > .05), supporting the validity of combining direct and indirect evidence. Pairwise comparisons showed that, relative to the negative control, both hUCMSCs-Exo and hAFSCs-Exo significantly reduced OARSI scores (Figure 2B). According to the SUCRA rankings (which reflect probabilistic ranking rather than definitive evidence of superiority), the cumulative ranking probabilities from highest to lowest were as follows: hAFSCs-Exo (SUCRA = 92.9%), hUCMSCs-Exo (SUCRA = 82.3%), BMSCs-Exo (SUCRA = 54.4%), hUSCs-Exo (SUCRA = 45.4%), ADMSCs-Exo (SUCRA = 42.9%), and DC-Exo (SUCRA = 28.7%), with the negative control having the lowest SUCRA value (3.5%) (Figure 2C). Funnel plots suggested potential publication bias and small-study effects (Figure 2D).

To explore the optimal transplantation dose, we performed dose-based network meta-analyses for BMSCs-Exo and hUCMSCs-Exo. For hUCMSCs-Exo, both high and low doses were significantly superior to the negative control, but the direct comparison between the two doses showed no statistically significant difference. SUCRA ranking further highlighted the advantage of the high dose, which had a substantially higher probability of being the best treatment (SUCRA = 93.2%) than the low dose (SUCRA = 56.8%). For BMSCs-Exo, both high and medium doses significantly reduced OARSI scores compared with negative controls, but no statistically significant difference in efficacy was observed between these two doses. The SUCRA results showed that the high dose (SUCRA = 83.4%) had a higher probability of being the optimal treatment than the medium dose (SUCRA = 66.4%). Asymmetric funnel plots indicated the possible presence of publication bias and small-study effects (Figure S3, see online supplementary material for a color version of this figure).

#### Type II collagen expression

Eighteen studies evaluated the effect of exosomes on type II collagen expression in the cartilage of rats with KOA. Conventional meta-analysis using a random-effects model (*I*^2^ = 84.7%, *P* < .0001) showed that exosome treatment significantly increased type II collagen expression (SMD = 3.41, 95% CI: 1.87 to 4.95). Subgroup analyses indicated significant differences across combinations of exosome source and dose (test for subgroup differences *P* < .0001). Within the same source, hUCMSCs-Exo significantly promoted expression at both low and high doses with comparable effects; for ADMSCs-Exo, the high-dose group showed a stronger effect than the low-dose group, whereas the medium-dose group showed no significant effect. Across the same dose level, effects differed by source: under low-dose conditions, SFB-Exo showed the largest promoting effect; under high-dose conditions, the pooled effect size for BMSCs-Exo was the most pronounced (Figure S4, see online supplementary material for a color version of this figure).

To identify the optimal exosome type for promoting type II collagen expression, we conducted network meta-analyses separately for low-, medium-, and high-dose groups. In the high-dose group (≥100 μg), 5 interventions were compared. Relative to the negative control, none of the exosome interventions showed a statistically significant promoting effect. However, the SUCRA rankings suggested that ADMSCs-Exo (SUCRA = 74.7%) and BMSCs-Exo (SUCRA = 76.3%) had relatively higher probabilities of being the most effective options, with similar superiority over the other types. The medium-dose analysis (40-100 μg) included 3 interventions. BMSCs-Exo demonstrated a significantly greater effect than negative controls, whereas ADMSCs-Exo did not differ significantly from negative controls. SUCRA rankings indicated that BMSCs-Exo had a high probability of being the best choice (SUCRA = 98.3%). The low-dose analysis (<40 μg) encompassed 5 interventions. The network meta-analysis showed that SFB-Exo exerted the most pronounced promotive effect compared with negative controls (SMD = 6.41, 95% CI: 2.35 to 10.47). According to the SUCRA rankings, SFB-Exo had the highest probability of being the optimal intervention (SUCRA = 99.2%), followed by hUCMSCs-Exo (SUCRA = 69.5%) and pExo (SUCRA = 46.5%). Tests of consistency indicated no significant inconsistency between direct and indirect evidence. Asymmetric funnel plots suggested potential publication bias and small-study effects (Figure S5, see online supplementary material for a color version of this figure).

To determine the optimal transplantation dose, we conducted dose-comparison network meta-analyses for ADMSCs-Exo, BMSCs-Exo, and hUCMSCs-Exo. For hUCMSCs-Exo, high and low doses showed comparable effects, although the high dose still had an advantage in ranking probability (SUCRA = 79.4%). For BMSCs-Exo, both high and medium doses exhibited significantly greater promotive effects than negative controls, although the direct comparison between these two doses did not reach statistical significance. SUCRA rankings suggested that the high dose might confer more pronounced benefit (SUCRA = 91.2%). For ADMSCs-Exo, none of the dose groups differed significantly from negative controls, but the SUCRA values indicated that the high dose had the highest relative probability of being the best option (SUCRA = 80.6%). Asymmetric funnel plots suggested publication bias and small-study effects (Figure S6, see online supplementary material for a color version of this figure).

#### Interleukin-1 beta

Twelve studies reported IL-1β. Conventional meta-analysis using a random-effects model (*I*^2^ = 56.3%, *P* = .0111) showed that exosome treatment significantly reduced IL-1β levels (SMD = −3.26, 95% CI: −4.08 to −2.44). Subgroup analyses suggested dose-related differences within the same source. For hUCMSCs-Exo, both low dose (SMD = −3.15) and high dose (SMD = −5.21) significantly reduced IL-1β, with a stronger effect at high dose; similarly, BMSCs-Exo was effective at both high dose (SMD = −5.10) and medium dose (SMD = −3.02), with a stronger effect at high dose. At the same dose level, efficacy also varied by source. In the low-dose group, except for SFB-Exo, hUCMSCs-Exo, M2D-Exo, and ADMSCs-Exo all showed significant inhibitory effects; in the medium-dose group, ADMSCs-Exo and BMSCs-Exo were effective; in the high-dose group, hUCMSCs-Exo, BMSCs-Exo, and M2D-Exo all showed significant anti-inflammatory effects (Figure S7, see online supplementary material for a color version of this figure).

To identify the most effective type of exosomes for reducing the inflammatory cytokine IL-1β, we conducted separate network meta-analyses for the low-, medium-, and high-dose groups. Under high-dose conditions (≥100 µg), BMSCs-Exo and hUCMSCs-Exo were compared; SUCRA rankings indicated that hUCMSCs-Exo had a higher probability of being the best treatment (SUCRA = 81.9%) than BMSCs-Exo (SUCRA = 68.1%). At medium doses (40-100 µg), ADMSCs-Exo and BMSCs-Exo were analyzed, and both interventions effectively reduced IL-1β levels. SUCRA rankings suggested that ADMSCs-Exo had a higher probability of being the optimal treatment (SUCRA = 80.8%) than BMSCs-Exo (SUCRA = 66.9%). At low doses (<40 µg), 4 types of exosomes—ADMSCs-Exo, M2D-Exo, SFB-Exo, and hUCMSCs-Exo—were compared. SUCRA rankings showed that M2D-Exo had the highest probability of being the best intervention (SUCRA = 89.4%), whereas the SUCRA values for hUCMSCs-Exo and SFB-Exo were 67.7% and 28.4%, respectively. Across all dose groups, no significant local or global inconsistency was detected, supporting the reliability of the pooled evidence; however, asymmetric funnel plots suggested potential publication bias and small-study effects (Figure S8, see online supplementary material for a color version of this figure).

To evaluate the inhibitory effects of different exosome doses on IL-1β expression, we then performed dose-comparison network meta-analyses for ADMSCs-Exo, BMSCs-Exo, and hUCMSCs-Exo. For hUCMSCs-Exo, both high and low doses showed significant efficacy, with SUCRA rankings indicating a higher probability for the high dose to be the best treatment (SUCRA = 95.6%). For BMSCs-Exo, both high and medium doses significantly reduced IL-1β levels, although the direct comparison between these two doses did not reach statistical significance. SUCRA rankings suggested that the high dose had a greater probability of being the optimal treatment (SUCRA = 91.4%) than the medium dose. For ADMSCs-Exo, both medium and low doses were significantly more effective than negative controls, with no significant difference between the two doses. The SUCRA values indicated that the medium dose had the highest probability of being the best option (SUCRA = 90.3%). Consistency tests for all analyses did not reveal significant local or global inconsistency, but asymmetric funnel plots indicated possible publication bias and small-study effects (Figure S9, see online supplementary material for a color version of this figure).

#### Publication bias assessment

Publication bias analysis based on type II collagen expression showed some asymmetry in the funnel plot. Egger’s test (intercept = 5.398, *P* < .001) and Begg’s test (Kendall’s tau = 4.366, *P* < .001) were both statistically significant, confirming publication bias. After correction using the trim-and-fill method, the pooled effect size decreased from SMD = 3.41 (95% CI: 1.87 to 4.95) to SMD = 1.26 (95% CI: 0.56 to 2.21). Although the estimated effect size was attenuated, the corrected 95% CI did not cross zero, indicating that the overall effect of exosomes in promoting type II collagen expression remained statistically significant (Figure S10, see online supplementary material for a color version of this figure).

## Discussion

This network meta-analysis systematically evaluated and compared the efficacy of exosome-based therapies for KOA in rat models under different treatment strategies. The central findings corroborate the overall therapeutic value of exosomes and further demonstrate that their efficacy is markedly influenced by cell source and transplantation dose. Conventional meta-analyses consistently showed that exosome treatment significantly improved cartilage structure (OARSI score), enhanced anabolic activity (type II collagen), and suppressed inflammation (IL-1β). Several plausible biological mechanisms have been proposed to explain these effects. For example, exosomes may deliver specific miRNAs and other bioactive cargos that modulate chondrocyte metabolic homeostasis: on the one hand, inhibiting pro-inflammatory pathways such as NF-κB/MAPK and downregulating matrix-degrading enzymes; on the other hand, activating anabolic pathways such as PI3K/Akt and TGF-β/Smad to promote matrix synthesis.^14–19^ Other studies have suggested that exosomes (eg, those enriched in miR-124) may directly target and reduce key inflammatory mediators such as IL-1β, thereby improving the joint microenvironment, and may inhibit chondrocyte apoptosis via regulation of pathways such as Bcl-2/Bax.^20–22^ These mechanistic hypotheses provide a possible molecular basis for the observed improvements in outcomes such as OARSI scores but require further validation in future studies.

One of the key findings of this study is the clear source dependence of efficacy. Based on the probabilistic SUCRA rankings, hAFSCs-Exo and hUCMSCs-Exo ranked highest for cartilage repair (OARSI score), and in the rat models included here, they outperformed exosomes derived from more common sources such as bone marrow and adipose tissue. This difference may arise from their distinct “molecular cargo” profiles. hAFSCs originate from an embryonic developmental milieu, and their exosomes are enriched in miRNAs associated with early morphogenesis and high proliferative potential, conferring prominent regenerative capacity.^23^^,^^24^ Human UCMSCs-Exo retain certain embryonic features combined with strong immunomodulatory properties and high expression of multiple growth factors, which may underlie their superior performance across several endpoints.^25^^,^^26^ By contrast, exosomes derived from adult tissues (eg, bone marrow and adipose) tend to carry molecular signatures oriented toward the maintenance of tissue homeostasis, with comparatively limited regenerative potential.^27^^,^^28^ This finding has implications for optimizing preclinical animal study design and informing translational strategy selection. However, it should be emphasized that substantial differences exist between rats and humans in joint anatomy, biomechanical environment, and disease course, and caution is warranted when extrapolating these results to clinical application.

In addition, the therapeutic effects showed a clear dose-dependent pattern. High-dose regimens (≥100 μg) tended to outperform medium- and low-dose regimens across the main outcomes, which may be related to the pharmacokinetic properties within the joint cavity. Given the relatively short half-life of exosomes, a sufficiently high dose is required to achieve an effective therapeutic concentration and to activate downstream signaling pathways.^29–31^ However, the dose–response relationship may not be linear and may exhibit a “plateau” effect. For example, with hUCMSCs-Exo, low doses already significantly enhanced type II collagen synthesis, whereas high doses showed a more pronounced advantage in terms of anti-inflammatory effects; ADMSCs-Exo, by contrast, may achieve an optimal balance at medium doses. This suggests that the optimal dose may need to be tailored according to the exosome source and the primary therapeutic goal (regeneration vs anti-inflammation). It is noteworthy that, although high doses generally ranked higher in the SUCRA-based ordering, this finding should be interpreted cautiously. In multiple direct comparisons, the differences between high and lower doses did not reach statistical significance. SUCRA rankings provide a probabilistic ordering that reflects the likelihood of a given dose being the best option within the current evidence network, but they do not constitute definitive evidence of superiority. Therefore, although the data suggest that higher doses may be more effective, the current direct-comparison evidence is insufficient to establish a conclusive dose–response relationship. This trend needs to be confirmed in future head-to-head dose-comparison studies.

The validity of our network meta-analysis rests on the transitivity assumption, which requires that the distributions of effect modifiers are comparable across different comparisons in the evidence network. Several potential effect modifiers vary considerably across the included studies, and these differences directly affect the credibility of indirect comparisons. Rat strains (Sprague–Dawley vs Wistar) may respond differently to identical exosome interventions due to genetic background variations. Osteoarthritis induction methods—primarily surgical (ACLT, medial meniscectomy) vs chemical (collagenase, monoiodoacetate)—create distinct pathological microenvironments: surgical models mimic post-traumatic degeneration, while chemical models involve more direct chondrotoxicity and acute inflammation.^32^ Exosome sources themselves constitute a major effect modifier, as the molecular cargo composition differs fundamentally between embryonic-derived (hAFSCs), perinatal (hUCMSCs), and adult (BMSCs, ADMSCs) tissues. Dosing schedules (single vs multiple injections) and the timing of outcome assessment (ranging from 2 to 12 weeks post-induction) capture different phases of disease evolution and repair. Measurement techniques for outcomes like type II collagen (immunohistochemistry with varied antibodies and quantification algorithms vs Western blot) produce systematically different effect magnitudes. When these factors are unevenly distributed across the evidence network, the transitivity assumption may be violated, leading to biased indirect estimates. Our node-splitting analysis did not detect significant global or local inconsistency, which provides some empirical support for transitivity. However, consistency tests have limited power to detect violations when the number of studies is modest. Therefore, readers should interpret the indirect comparisons and SUCRA rankings with the understanding that unmeasured or imbalanced effect modifiers may influence the results.

This study builds on the existing literature and provides an important advance. Previous meta-analyses have often pooled data from different species or focused on a limited range of exosome sources.^11–13^ In contrast, the present study was strictly restricted to rat models, thereby reducing heterogeneity, and for the first time used network meta-analysis to systematically compare multiple combinations of sources, doses, and administration routes, providing probabilistic rankings of interventions. Nevertheless, the conclusions of this study should be interpreted cautiously in light of several limitations.

First, the methodological quality of the included studies is a key constraint on the strength of the evidence. According to the SYRCLE risk-of-bias assessment, most studies had unclear risk of bias in critical domains such as random sequence generation and allocation concealment, suggesting potential selection and performance biases that may compromise the reliability of the effect estimates. This limitation directly reduces the overall certainty of the evidence in this review. Second, there was substantial between-study heterogeneity. In the conventional meta-analyses, *I*^2^ values reached 84.7% for type II collagen expression, 66.9% for OARSI scores, and 56.3% for IL-1β, indicating moderate to high heterogeneity. Likely sources include differences in model induction methods (eg, anterior cruciate ligament transection vs other surgical or chemical induction approaches), exosome dosing regimens (eg, single vs multiple injections and injection intervals), timing of outcome assessment (eg, different postoperative time points), and measurement techniques (eg, immunohistochemistry vs Western blot) and quantification methods. These factors may all influence the estimated effect sizes and reduce confidence in the pooled results. Although we attempted to explore sources of heterogeneity through subgroup analyses and network meta-analyses, the limited number of included studies precluded more in-depth meta-regression. Third, publication bias represents a particularly important limitation of this work. The asymmetry of funnel plots and the significance of Egger’s/Begg’s tests clearly indicate the presence of publication bias. Trim-and-fill analysis showed that the pooled effect size for type II collagen expression decreased markedly from SMD = 3.41 to 1.26, strongly suggesting that the original effect size was substantially overestimated. This overestimation directly lowers the certainty of any treatment effect estimates derived from the current data. In addition, the network meta-analyses relied predominantly on indirect comparisons, with limited direct head-to-head evidence; SUCRA rankings should therefore be viewed as probabilistic signals rather than definitive conclusions. Finally, all evidence in this review was derived from rat models. Although rats are widely used in osteoarthritis research, there are important differences from humans in knee joint anatomy, cartilage thickness, biomechanical loading, and the natural history of disease. The efficacy patterns and ranking results observed here cannot be assumed to translate directly into clinical benefit, and substantial caution is required when extrapolating these findings to humans. In sum, interpretation of exosome efficacy must fully account for the impact of heterogeneity, publication bias, and methodological limitations on the strength of the evidence so as to avoid overly optimistic conclusions. Future high-quality studies with rigorous methodology and long-term follow-up are needed to support clinical translation.

## Conclusion

In summary, meta-analytic evidence from existing rat models suggests that exosomes may effectively improve cartilage structure, anabolic activity, and inflammatory status in KOA. According to the probabilistic SUCRA rankings, hAFSCs-Exo and hUCMSCs-Exo demonstrated a relatively higher probability of cartilage repair potential under the conditions of this study, and high-dose strategies showed a generally more favorable probabilistic trend. Even so, most direct comparisons between high and medium or low doses did not reach statistical significance; the dose–response relationship should therefore be regarded as an exploratory trend rather than a definitive conclusion.

It is important to emphasize that, because the included studies commonly had unclear risk of bias in key methodological domains such as randomization and allocation concealment, the overall certainty of the evidence is low. Moreover, the above findings are constrained by the methodological quality of the original studies, substantial between-study heterogeneity, publication bias, and relatively small sample sizes and should be regarded as exploratory, providing hypotheses and directions for future research. In particular, the presence of publication bias and the results of the trim-and-fill analyses indicate that the current effect sizes may be substantially overestimated, and the certainty of the treatment effect estimates is consequently weakened. Therefore, methodologically rigorous preclinical studies with long-term follow-up are needed, and, when conditions permit, clinical trials should be conducted to verify the safety and efficacy of these potentially optimized strategies.

## Supplementary Material

szag069_Supplementary_Data

## Data Availability

The data underlying this article will be shared upon reasonable request to the corresponding author.
