# Supplementary material for: Exosome therapy for knee osteoarthritis: a network meta-analysis based on rat models
Source: Stem Cells Transl Med. 2026 Aug 18;15(9):szag069. doi: 10.1093/stcltm/szag069 (PMC13485348; doi:10.1093/stcltm/szag069)
Supplement: Supplementary file 1 [file szag069_supplementary_data.docx]

**Supplemental Files**

**Title: Exosome Therapy for Knee Osteoarthritis: A Network Meta-Analysis Based on Rat Models**

**Running title: Exosome Therapy for Knee Osteoarthritis**

**Author:** Yixiang Zhao^1^, Haochuan You^1^, Xiuyuan Wang^1^, Xiping Dou^1^, Jiangying Zhang^1^, Yuzhe He^1^, Lei Chen^1^, Fei Huang^1^, Yayi Xia^1^

**Affiliation:** ^1^Department of Orthopedics, Lanzhou University Second Hospital, , Lanzhou, Gansu Province, China

**Corresponding author:** Yayi Xia, Ph.D., Professor, The Second Hospital of Lanzhou University, Lanzhou 730030, China. Email: xiayy@lzu.edu.cn; Tel.: +86 13893123864.

**Risk of Bias Assessment**

The SYRCLE risk of bias tool was used to systematically assess bias across ten key domains: random sequence generation, allocation concealment, baseline characteristic balance, random housing, blinding (researchers/animal handlers), outcome assessment blinding, incomplete data reporting, selective outcome reporting, other biases (such as conflicts of interest or funding support), and sample size calculation. Each domain was rated as "low risk of bias (yes)," "high risk of bias (no)," or "unclear (insufficient information)." Disagreements were resolved by consensus or through consultation with a third researcher.

**Data Extraction from Figures and Handling of Statistical Parameters**

For studies that reported outcome data only in graphical form (e.g., bar charts with error bars), two investigators independently extracted numerical values using WebPlotDigitizer (version 4.6). Each graph was calibrated using its axes, and each bar or point was digitized three times to obtain an average value. Discrepancies between the two investigators exceeding 10% prompted re-extraction and discussion. When studies reported standard error of the mean (SEM) instead of standard deviation (SD), we converted SEM to SD using the formula SD = SEM × √n, where n is the group sample size. For studies with multiple post-treatment time points, we extracted data from the time point closest to the end of the observation period in each study, typically 4, 6, or 8 weeks after osteoarthritis induction. When a study contained more than two intervention arms (e.g., two different exosome sources or doses), we split the shared control group among the comparisons to avoid double-counting, following Cochrane Handbook recommendations.

**Statistical Analysis**

In traditional head-to-head meta-analysis, all outcome measures were continuous variables. Standardized mean differences (SMD) with 95% confidence intervals (CIs) were used as the effect size to eliminate differences in measurement methods and units across studies. Heterogeneity was assessed using Cochran's Q test (significance level set at α=0.10) and the I² statistic. If I² ≤ 50% and the Q test P ≥ 0.10, heterogeneity was considered acceptable, and a fixed-effect model was used for pooled analysis. If I² > 50% or Q test P < 0.10, significant heterogeneity was present, and a random-effects model (restricted maximum likelihood estimation of between-study variance τ²) was used. Subgroup analyses were performed to explore sources of heterogeneity. Preset subgroup analyses included exosome source, transplantation dose, and route. A funnel plot was used for visual assessment of publication bias for the most frequently reported outcome, followed by Egger's linear regression test and Begg's rank correlation test (P < 0.05 indicating publication bias). If publication bias was detected, the trim-and-fill method was used for correction, assessing the impact of bias on the pooled effect size.

In the frequentist network meta-analysis, a consistency model was first applied, assuming that direct and indirect comparisons yield consistent results. In cases of inconsistency, an inconsistency model was used for correction, and the consistency of network evidence was evaluated through node-splitting and loop inconsistency tests (P < 0.05 indicating significant inconsistency). Effect sizes for direct, indirect, and pooled comparisons (SMD with 95% CI) were calculated to clarify relative efficacy differences across different exosome sources, doses, and routes. Surface Under the Cumulative Ranking Curve (SUCRA) values were used to rank the efficacy of each intervention, with higher SUCRA values indicating better therapeutic efficacy. Heterogeneity was evaluated using τ² to estimate within-study and between-study heterogeneity. If heterogeneity was excessive, subgroup analyses or random-effects models were employed for further pooling.

All statistical analyses were performed using R statistical software (version 4.4.2) and STATA 16.0. For conventional meta-analysis, we used the ‘meta’ package in R (function ‘metacont’) with the inverse-variance method and restricted maximum-likelihood estimator for random-effects models. For network meta-analysis, we used the ‘netmeta’ package (function ‘netmeta’) with a frequentist graph-theoretical approach. The heterogeneity variance (τ²) was estimated using the restricted maximum-likelihood method. Inconsistency was evaluated globally using the design-by-treatment interaction test and locally using the node-splitting method implemented in the ‘netsplit’ function. SUCRA values were calculated using the ‘netrank’ function. In STATA, we performed additional publication bias diagnostics using the ‘metabias’ command (Egger’s test) and the ‘metatrim’ command (trim-and-fill). All tests were two-sided, and P < 0.05 was considered statistically significant. A 95% CI not containing zero indicated a significant therapeutic effect.

We did not perform additional sensitivity analyses excluding studies with very small sample sizes or unclear risk of bias. Animal studies in this field typically enroll 6–12 rats per group, and sample sizes are uniformly modest. Excluding studies based on sample size would discard most included trials without meaningful gain in precision. Similarly, excluding studies with unclear risk of bias—which affected nearly all included trials in domains such as random sequence generation and allocation concealment—would reduce the evidence network to an extent that many nodes would become disconnected and several comparisons would rely on single studies. We therefore retained all eligible studies and instead transparently reported the risk-of-bias assessment and discussed its implications in the main text.

Table 1. Search strategy

**1.PubMed**

#1: "exosome*"[Title/Abstract] OR "exosomes"[Title/Abstract] OR "exosomal"[Title/Abstract] OR "extracellular vesicle"[Title/Abstract] OR "extracellular vesicles"[Title/Abstract] OR "extracellular particle"[Title/Abstract] OR "extracellular particles"[Title/Abstract] OR "microvesicle"[Title/Abstract] OR "microvesicles"[Title/Abstract] OR "Shedding Microvesicle"[Title/Abstract] OR "Shedding Microvesicles"[Title/Abstract] OR "Secretory Vesicle"[Title/Abstract] OR "Secretory Vesicles"[Title/Abstract] OR "Cell-Derived Microparticle"[Title/Abstract] OR "Cell-Derived Microparticles"[Title/Abstract] OR "microbubble"[Title/Abstract] OR "microbubbles"[Title/Abstract] OR "apoptotic body"[Title/Abstract] OR "apoptosis bodies"[Title/Abstract]

#2: "Exosomes"[MeSH Terms] OR "Extracellular Vesicles"[MeSH Terms] OR "Cell-Derived Microparticles"[MeSH Terms] OR "Microbubbles"[MeSH Terms]

#3: #1 OR #2

#4: "osteoarthritis"[Title/Abstract] OR "knee osteoarthritis"[Title/Abstract] OR "KOA"[Title/Abstract]

#5: "osteoarthritis, knee"[MeSH Terms]

#6: #4 OR #5

#7: #3 AND #6

**2. WOS**

(TS=("exosome*" OR "exosomes" OR "exosomal" OR "extracellular vesicle" OR "extracellular vesicles" OR "extracellular particle" OR "extracellular particles" OR "microvesicle" OR "microvesicles" OR "Shedding Microvesicle" OR "Shedding Microvesicles" OR "Secretory Vesicle" OR "Secretory Vesicles" OR "Cell-Derived Microparticle" OR "Cell-Derived Microparticles" OR "microbubble" OR "microbubbles" OR "apoptotic body" OR "apoptosis bodies")) AND TS=("osteoarthritis" OR "knee osteoarthritis" OR "KOA") AND (Refinement basis:Literature Type: Thesis or Review Paper or Other or Online Publication or Clinical Trial)

**3. Embase**

#1: 'exosome*':ti,ab,kw OR 'exosomes':ti,ab,kw OR 'exosomal':ti,ab,kw OR 'extracellular vesicle':ti,ab,kw OR 'extracellular vesicles':ti,ab,kw OR 'extracellular particle':ti,ab,kw OR 'extracellular particles':ti,ab,kw OR 'microvesicle':ti,ab,kw OR 'microvesicles':ti,ab,kw OR 'shedding microvesicle':ti,ab,kw OR 'shedding microvesicles':ti,ab,kw OR 'secretory vesicle':ti,ab,kw OR 'secretory vesicles':ti,ab,kw OR 'cell-derived microparticle':ti,ab,kw OR 'cell-derived microparticles':ti,ab,kw OR 'microbubble':ti,ab,kw OR 'microbubbles':ti,ab,kw OR 'apoptotic body':ti,ab,kw OR 'apoptosis bodies':ti,ab,kw

#2: 'exosome'/exp

#3: 'exosomes'/exp

#4: 'extracellular vesicle'/exp

#5: 'microvesicle'/exp

#6: 'secretory vesicle'/exp

#7: 'cell-derived microparticle'/exp

#8: 'microbubble'/exp

#9: 'apoptotic body'/exp

#10: #1 OR #2 OR #3 OR #4 OR #5 OR #6 OR #7 OR #8 OR #9

#11: 'osteoarthritis':ti,ab,kw OR 'knee osteoarthritis':ti,ab,kw OR 'koa':ti,ab,kw

#12: 'osteoarthritis'/exp

#13: 'knee osteoarthritis'/exp

#14: #11 OR #12 OR #13

#15: #10 AND #14

#16: #15 AND ('article'/it OR 'article in press'/it OR 'clinical trial'/it OR 'preprint'/it OR 'review'/it OR 'short survey'/it)

**4. Scopus**

TITLE-ABS-KEY ( "exosome*" OR "exosomes" OR "exosomal" OR "extracellular vesicle" OR "extracellular vesicles" OR "extracellular particle" OR "extracellular particles" OR "microvesicle" OR "microvesicles" OR "Shedding Microvesicle" OR "Shedding Microvesicles" OR "Secretory Vesicle" OR "Secretory Vesicles" OR "Cell-Derived Microparticle" OR "Cell-Derived Microparticles" OR "microbubble" OR "microbubbles" OR "apoptotic body" OR "apoptosis bodies" ) AND TITLE-ABS-KEY ( "osteoarthritis" OR "knee osteoarthritis" OR "KOA" ) AND ( LIMIT-TO ( DOCTYPE , "ar" ) OR LIMIT-TO ( DOCTYPE , "re" ) OR LIMIT-TO ( DOCTYPE , "sh" ) )

Table 2. Baseline characteristics of the included studies.

| **No.** | **Author** | **Year** | **Country** | **Research type** | **Animal Characteristics** | | | | **Sample Size** | **Modeling Method** | **Exosome** | | **Transplant Route** | **Transplant Dose** | Control Group |
| --- | --- | --- | --- | --- | --- | --- | --- | --- | --- | --- | --- | --- | --- | --- | --- |
|  |  |  |  |  | **Species** | **Gender** | **Weight** | **Age** | **Experimental/Control Group** |  | **Type** | **Source** |  |  |  |
| 3 | Yin | 2023 | China | RCT | SD rats | Male | 250 ± 50 g | 6–8 weeks | 6/6 | Creation of osteochondral defects in the femoral trochlea of the knee joint (diameter 2 mm, depth 1.5 mm) | M2D-Exo | IL-4 induced M2D from bone marrow-derived rats | Intra-articular injection | 5 μg | PBS |
| 4 | Chen | 2022 | China | RCT | SD rats | Male | / | 8 weeks | 6/6 | ACLT | hUCMSCs-Exo | Human UCMSCs | Intra-articular injection | 30 μg | PBS |
| 5 | Zhang | 2022 | China | RCT | SD rats | Female | / | 4 weeks | 10/10 | Transection of the anterior cruciate ligament + Medial meniscus resection | BMSCs-Exo | Human BMSCs | Intra-articular injection | 100 μg | PBS |
| 7 | Zhang | 2025 | China | RCT | SD rats | Male | 180-200 g | / | 6/6 | Anterior cruciate ligament transection combined with medial meniscus resection (ACLT + MMx) | SFB-Exo | SFBisolated from the synovial tissue of SD rats | Intra-articular injection | 20 μg | PBS |
| 8 | Liao | 2021 | China | RCT | SD rats | Male | 250±20 g | 8 weeks | 7/6 | ACLT + MMx | BMSCs-Exo | BMSCs extracted from rat bone marrow | Intra-articular injection | 40 μg | Physiological saline |
| 9 | Meng | 2023 | China | RCT | SD rats | Male | 200±10 g | 6 weeks | 10/10 | Intra-articular injection of sodium iodoacetate solution for 7 consecutive days (chemical induction model) | ADMSCs-Exo | ADMSCs isolated from the adipose tissue of SD rats | Intra-articular injection | 40 μg | Physiological saline |
| 11 | Kang | 2025 | China | RCT | SD rats | Male | 180-210 g | / | 6/6 | Surgical instability of the medial meniscus | ADMSCs-Exo | ADMSCs isolated from the epididymal fat of SD rats | Intra-articular injection | 40 μg | Physiological saline |
| 12 | Rong | 2021 | China | RCT | SD rats | Female | 200–250 g | 7–8 weeks | 10/10 | ACLT + MMx | BMSCs-Exo | BMSCs extracted from rat bone marrow | Intra-articular injection | 200 μg | PBS |
| 14 | Xu | 2021 | China | RCT | SD rats | Male | 200–220 g | Adult | 6/6 | Intra-articular injection of 8% sodium iodoacetate | BMSCs-Exo | BMSCs from rats | Intra-articular injection | 40 μg | PBS |
| 17 | Li | 2023 | China | RCT | SD rats | Male | 220 g | 10 weeks | 6/6 | Anterior cruciate ligament transection combined with medial meniscus resection | BMSCs-Exo | BMSCs extracted from rat bone marrow | Intra-articular injection | 100 μg | PBS |
| 20 | Li | 2021 | China | RCT | Wistar rats | Female | 180 ± 20 g | Adult | 6/6 | Full-thickness cartilage defect model, diameter 1 mm, depth 0.2 mm | ADMSCs-Exo | ADMSCs isolated from the adipose tissue of SD rats | Intra-articular injection | 100 μg | Blank control |
| 23 | Kang | 2025 | China | RCT | SD rats | Male | 150 ± 10 g | 6 weeks | 10/10 | Anterior cruciate ligament transection | ADMSCs-Exo | ADMSCs from rats | Intra-articular injection | 5 μg | PBS |
| 29 | Huang | 2023 | China | RCT | SD rats | Male | / | 10–12 weeks | 10/11 | MCLT + MMT (Medial collateral ligament transection + Medial meniscus tear) | pExo | Healthy full-term human placental tissue | Intra-articular injection | 20 μg | Physiological saline |
| 30 | He | 2020 | China | RCT | SD rats | Male | / | 10 weeks | 8/8 | Intra-articular injection of sodium iodoacetate | BMSCs-Exo | BMSCs extracted from rat bone marrow | Intra-articular injection | 40 μg | Physiological saline |
| 31 | El-Din | 2024 | Egypt | RCT | Wistar rats | Male | 150–250 g | 8–10 weeks | 8/8 | Intra-articular injection of sodium iodoacetate | BMSCs-Exo | BMSCs extracted from rat bone marrow | Intra-articular injection | 100 μg | Blank control |
| 32 | Zhang | 2025 | Singapore | RCT | SD rats | Female | 220–250 g | 8 weeks | 6/6 | ACLT | BMSCs-Exo | Human BMSCs | Intra-articular injection | 120 μg | PBS |
| 34 | Zhou | 2021 | China | RCT | SD rats | Male | 200–250 g | / | 5/5 | Anterior cruciate ligament transection + Medial meniscus resection | SFB-Exo | SFB isolated from the synovial tissue of SD rats | Intra-articular injection | 20 μg | PBS |
| 36 | Esmaeili | 2022 | Iran | RCT | Wistar rats | Male | 230-260 g | Adult | 5/5 | Single intra-articular injection of sodium iodoacetate for induction | BMSCs-Exo | BMSCs from rabbits | Intra-articular injection | 50 μg | PBS |
| 39 | Qin | 2025 | China | RCT | SD rats | Male | 200–220 g | 6 weeks | 8/8 | Transection of the medial meniscus + Transection of the anterior cruciate ligament | hUCMSCs-Exo | Human UCMSCs | Intra-articular injection | 350 μg | PBS |
| 43 | Zavatti | 2020 | Italy | RCT | CD rats（Charles River） | / | / | 8 weeks | 8/4 | Monoiodoacetate (MIA) intra-articular injection to induce osteoarthritis | hAFSCs-Exo | Extracted from the supernatant of human amniotic stem cell cultures | Intra-articular injection | 100 μg | Physiological saline |
| 44 | Liu | 2025 | China | RCT | SD rats | Female | / | 9 weeks | 6/6 | Anterior cruciate ligament transection combined with medial meniscus instability surgery | hUSCs-Exo | Human urine-derived stem cells | Intra-articular injection | 100 μg | PBS |
| 45 | Zhang | 2024 | China | RCT | SD rats | Male | 250 ± 20 g | 8 weeks | 6/6 | Intra-articular injection of sodium iodoacetate to induce OA (osteoarthritis) | BMSCs-Exo | BMSCs extracted from rat bone marrow | Intra-articular injection | 75 μg | Physiological saline |
| 46 | Liang | 2020 | China | RCT | SD rats | Male | 200–220 g | / | 5/5 | Medial meniscus instability surgery | DC-Exo | Dendritic cells | Intra-articular injection | 100 μg | PBS |
| 48 | Pan | 2021 | China | RCT | SD rats | Male | / | 10 weeks | 4/4 | Intra-articular injection of type II collagenase | BMSCs-Exo | Human BMSCs | Intra-articular injection | 40 μg | PBS |
| 52 | Tang | 2021 | China | RCT | SD rats | Female | / | 8 weeks | 6/6 | Anterior cruciate ligament transection | hUCMSCs-Exo | Human UCMSCs | Intra-articular injection | 30 μg | PBS |
| 55 | Kong | 2023 | China | RCT | SD rats | / | / | 8 weeks | 3/3 | Medial meniscus instability surgery | SMSC-Exo | SMSCs isolated from primary cultures of rats | Intra-articular injection | 100 μg | Physiological saline |
| 58 | Li | 2023 | China | RCT | SD rats | / | 230–280 g | / | 5/5 | Intra-articular injection of monoiodoacetate | ADMSCs-Exo | Human hADSCs | Intra-articular injection | 100 μg | Physiological saline |

Supplementary Figure 1. PRISMA flow diagram.

Supplementary Figure 2. Risk-of-bias assessment results.

Supplementary Figure 3. Dose–response comparison of hUCMSCs-Exo and BMSCs-Exo on OARSI scores: (A) network plot for hUCMSCs-Exo; (B) forest plot for hUCMSCs-Exo; (C) SUCRA rankings for hUCMSCs-Exo; (D) comparison-adjusted funnel plot for hUCMSCs-Exo; (E) network plot for BMSCs-Exo; (F) forest plot for BMSCs-Exo; (G) SUCRA rankings for BMSCs-Exo; (H) comparison-adjusted funnel plot for BMSCs-Exo.

Supplementary Figure 4. Conventional meta-analysis of type II collagen expression.

Supplementary Figure 5. Comparison of different exosome types in improving type II collagen expression at high, medium, and low transplantation doses (A, evidence network at high dose; B, forest plot at high dose; C, SUCRA ranking at high dose; D, comparison-adjusted funnel plot at high dose; E, evidence network at medium dose; F, forest plot at medium dose; G, SUCRA ranking at medium dose; H, comparison-adjusted funnel plot at medium dose; I, evidence network at low dose; J, forest plot at low dose; K, SUCRA ranking at low dose; L, comparison-adjusted funnel plot at low dose).

Supplementary Figure 6. Dose–response comparison of hUCMSCs-Exo, BMSCs-Exo, and ADMSCs-Exo on type II collagen expression: (A) network plot for hUCMSCs-Exo; (B) forest plot for hUCMSCs-Exo; (C) SUCRA rankings for hUCMSCs-Exo; (D) comparison-adjusted funnel plot for hUCMSCs-Exo; (E) network plot for BMSCs-Exo; (F) forest plot for BMSCs-Exo; (G) SUCRA rankings for BMSCs-Exo; (H) comparison-adjusted funnel plot for BMSCs-Exo; (I) network plot for ADMSCs-Exo; (J) forest plot for ADMSCs-Exo; (K) SUCRA rankings for ADMSCs-Exo; (L) comparison-adjusted funnel plot for ADMSCs-Exo.

Supplementary Figure 7. Conventional meta-analysis of IL-1β expression.

Supplementary Figure 8. Effects of exosome type on IL-1β reduction at high, medium, and low doses: high dose—(A) network plot; (B) forest plot; (C) SUCRA rankings; (D) comparison-adjusted funnel plot; medium dose—(E) network plot; (F) forest plot; (G) SUCRA rankings; (H) comparison-adjusted funnel plot; low dose—(I) network plot; (J) forest plot; (K) SUCRA rankings; (L) comparison-adjusted funnel plot.

Supplementary Figure 9. Dose–response comparison of hUCMSCs-Exo, BMSCs-Exo, and ADMSCs-Exo on IL-1β expression: (A) network plot for hUCMSCs-Exo; (B) forest plot for hUCMSCs-Exo; (C) SUCRA rankings for hUCMSCs-Exo; (D) comparison-adjusted funnel plot for hUCMSCs-Exo; (E) network plot for BMSCs-Exo; (F) forest plot for BMSCs-Exo; (G) SUCRA rankings for BMSCs-Exo; (H) comparison-adjusted funnel plot for BMSCs-Exo; (I) network plot for ADMSCs-Exo; (J) forest plot for ADMSCs-Exo; (K) SUCRA rankings for ADMSCs-Exo; (L) comparison-adjusted funnel plot for ADMSCs-Exo.

Supplementary Figure10. Publication bias assessment results (A, funnel plot; B, Egger’s test; C, Begg’s test; D, trim-and-fill results).
